# The Evolving Interplay among Abundant Adipokines in Patients with Hepatitis C during Viral Clearance

**DOI:** 10.3390/nu9060570

**Published:** 2017-06-02

**Authors:** Ming-Ling Chang, Tsung-Hsing Chen, Chen-Ming Hsu, Cheng-Hui Lin, Cheng-Yu Lin, Chia-Jung Kuo, Shu-Wei Huang, Chun-Wei Chen, Hao-Tsai Cheng, Chau-Ting Yeh, Cheng-Tang Chiu

**Affiliations:** 1 Liver Research Center, Division of Hepatology, Department of Gastroenterology and Hepatology, Chang Gung Memorial Hospital, Taoyuan 33305, Taiwan; itochenyu@gmail.com (T.-H.C.); hsu3060e@cloud.cgmh.org.tw (C.-M.H.); linchehui@cloud.cgmh.org.tw (C.-H.L.); 8805035@gmail.com (C.-Y.L.); m7011@cloud.cgmh.org.tw (C.-J.K.); huangshuwei@hotmail.com (S.-W.H.); 8902088@cloud.cgmh.org.tw (C.-W.C.); hautai@cloud.cgmh.org.tw (H.-T.C.); chauting@cloud.cgmh.org.tw (C.-T.Y.); ctchiu0508@gmail.com (C.-T.C.); 2 Department of Medicine, College of Medicine, Chang Gung University, Taoyuan 33305, Taiwan

**Keywords:** HCV, SVR, leptin, adiponectin, PAI-1, HOMA-IR

## Abstract

How hepatatitis C virus (HCV) infection affects the interplay among abundant adipokines in the host remains unclear. A prospective study was conducted with 450 consecutive genotype 1 (G1) and G2 HCV patients who completed a course of anti-HCV therapy and underwent pre-therapy and 24-week post-therapy surveys to assess various profiles and levels of abundant adipokines, including leptin, adiponectin, and plasminogen activator inhibitor-1 (PAI-1). Before anti-HCV therapy, multivariate analyses showed gender to be associated with leptin and adiponectin levels, and BMI with leptin and PAI-1 levels. Among patients with a sustained virological response (SVR, *n* = 372), associations at 24 weeks post-therapy were as follows: gender and BMI with all adipokine levels; hepatic steatosis and aspartate aminotransferase to platelet ratio index with adiponectin levels; and HOMA-IR and HCV genotype with PAI-1 levels. Paired *t*-tests revealed increased post-therapeutic PAI-1 levels in G1 SVR patients and decreased adiponectin levels in all SVR patients compared to pre-therapeutic levels. HCV infection may obscure associations between abundant adipokines and metabolic/hepatic profiles. In SVR patients, a higher hierarchical status of PAI-1 versus adiponectin in affecting glucose metabolism was noted at 24 weeks post-therapy. Such genotype-non-specific adiponectin decreases and G1-specific PAI-1 increases warrant careful follow-up of HCV patients after SVR according to viral genotype.

## 1. Introduction

Hepatitis C virus (HCV), a human pathogen responsible for acute and chronic liver disease, is classified into seven genotypes [1] and infects an estimated 150 million individuals worldwide [2]. In addition to liver cirrhosis and hepatocellular carcinoma, the virus causes metabolic alterations, including dyslipidemia, hepatic steatosis, obesity, insulin resistance (IR), and type 2 diabetes [2,3]. Much of the HCV life cycle is closely associated with lipid metabolism [2,3]. Moreover, both the direct effects of HCV on hepatocytes and the indirect mechanisms that involve extrahepatic organs influence insulin signaling [2,3].

Adipose tissue has emerged as an important endocrine organ due to its release of adipokines [4], which are biologically-active polypeptides that include the adipose tissue-specific adipokines leptin and adiponectin and non-adipose tissue-specific adipokines, such as plasminogen activator inhibitor-1 (PAI-1) [5]. With circulating concentrations that reach approximately 3–30 μg/mL, adiponectin is the most abundant adipokine [6]. Both adiponectin and leptin are abundant in subcutaneous fat [7], and high levels of PAI-1 are found in the extracellular matrix [8]. Thus, leptin, adiponectin and PAI-1 are abundant adipokines stable in the circulation and may regulate body lipid and glucose metabolism via the adipoinsular axis [9]. For example, leptin plays a crucial role in maintaining glucose metabolism [10], and adiponectin protects against obesity-related complications [11] by enhancing fatty acid oxidation and insulin sensitivity [12]. However, increased visceral adipose tissue reduces the biological activity of leptin and the abundance of circulating adiponectin [13]; the former is pro-inflammatory and positively associated with total body fat [14], whereas the latter is anti-inflammatory and negatively associated with hepatic steatosis and obesity [15]. Overall, circulating leptin levels are increased and adiponectin levels decreased in obese subjects [16]. Both leptin and adiponectin are reliable markers of many diseases, including lung [17] and cardiovascular diseases [18]. In addition, PAI-1 levels are increased in patients with metabolic syndrome [19], obesity, IR [20], non-alcoholic fatty liver disease [5] and inflammatory disease [21]. Moreover, PAI-1 is a central regulator of the fibrinolytic system [22] and can indicate a risk of developing cardiovascular disease [23]. In general, circulating leptin and adiponectin levels are positively associated with the female gender [16], whereas PAI-1 levels are positively associated with the male gender [24]. Together, levels of these adipokines indicate the degree of body fat accumulation, metabolic homeostasis, and sexual dimorphism, as well as the risk of developing the aforementioned diseases, particularly cardiometabolic diseases [10,11,12,13,14,15,16,17,18,19,20,21,22,23,24].

Since both HCV infection and adipokines are critically involved in metabolism, their potential relationship has attracted much research attention, with conflicting results [2]. In addition to the pleiotropic functions of adipokines described above, the lack of clarity regarding relationships is primarily due to variability among individuals, which is difficult to completely eliminate from case-controlled, retrospective, or prospective studies with a limited number of adjustments for confounders. Thus, our previous studies involved patients with chronic hepatitis C (CHC) who achieved a sustained virological response (SVR) served as their own least biased controls before therapy. Our results showed that after adjusting for crucial confounders, leptin and complement component 3 (C3) possibly maintain immune and metabolic homeostasis through associations with C4 and total cholesterol (TC), with unchanged levels after HCV clearance [25]. Within 24 weeks after anti-HCV therapy, SVR patients showed increasing PAI-1 levels with higher cardiovascular risk [24]. Additionally, we observed an evolving relationship between adiponectin levels and insulin sensitivity in CHC patients during viral clearance [26]. Although HCV infection is treatable using potent direct-acting antiviral agents [2], the interplay among the abundant adipokines produced in CHC patients achieving viral clearance has remained unclear. Furthermore, this process may be crucial for cardiometabolic homeostasis in these patients. Therefore, we sought to elucidate this process by conducting a prospective study to analyze HCV RNA levels as well as hepatic, metabolic, and abundant adipokine profiles in CHC patients before and after anti-HCV therapy.

## 2. Materials and Methods

### 2.1. Patients

The study group contained subjects 18 years or older with CHC, which was defined as the presence of documented HCV antibody positivity and detectable HCV RNA levels for at least 24 weeks. Subjects with human immunodeficiency virus or hepatitis B virus infection, hemochromatosis or malignancy and recipients of solid organ transplants were excluded.

### 2.2. Study Design

A total of 450 CHC (234 genotype 1 (G1) and 216 G2) patients were consecutively recruited at a tertiary referral center between July 2010 and August 2015. According to a response-guided therapeutic protocol, all patients received anti-HCV therapy with pegylated interferon alpha-2b (1.5 μg/kg/week) and ribavirin (800–1400 mg/day) [24,25,26,27]. HCV RNA levels were measured using a COBAS Amplicor (ver. 2.0, Roche Diagnostics, Tokyo, Japan), and the HCV genotype was determined using the InoLipa method (COBAS AmpliPrep/COBAS TaqMan HCV Test, Roche Diagnostics, Tokyo, Japan). Single-nucleotide polymorphisms (SNPs) in interferon-λ 3 (IFNL3; rs12979860) were assessed using genomic DNA, as previously described [24,25,26,27,28,29]. Patients were evaluated under fasting conditions two weeks prior to, and 24 weeks after, the end of therapy to determine the following: body weight; body mass index (BMI); HCV RNA, TC, triglyceride (TG), alanine aminotransferase (ALT), leptin, adiponectin, and PAI-1 levels; homeostasis model assessment-estimated insulin resistance (HOMA-IR)(fasting insulin (μU/mL)×fasting glucose (mmol/L)/22.5) score; and the aspartate aminotransferase (AST)-to-platelet ratio index (APRI) [30]. The leptin/adiponectin (L/A) ratio was also assessed. To monitor hepatic steatosis and cirrhosis, abdominal ultrasound analyses were performed for every patient before therapy and every six months thereafter. SVR was defined as an undetectable HCV RNA level at 24 weeks after therapy completion.

### 2.3. Adipokine Enzyme-Linked Immunosorbent Assays (ELISAs)

Serum leptin (male: 2.205–11.149 ng/mL; female 3.877–77.273 ng/mL), adiponectin (0.865–21.424 µg/mL), and PAI-1 (0.98–18.7 ng/mL) levels were assayed according to the manufacturer’s protocols (R and D Systems, Minneapolis, MN, USA).

### 2.4. Biochemistry

Serum biochemistry, HCV RNA measurement and genotype evaluation were performed using routine automated techniques in the clinical pathology or liver research laboratories of the hospital.

### 2.5. Statistical Analysis

All statistical analyses were performed using either Statistical Package for the Social Sciences (SPSS ver. 21.0, SPSS Inc., Chicago, IL, USA) or MedCalc (MedCalc ver. 12.4, MedCalc Software Corp., Brunswick, ME, USA) software. Continuous variables are presented as means ± standard deviations (SDs), and categorical variables are presented as frequencies and percentages. Continuous variables were analyzed using Student’s *t*-test and categorical variables using the chi-squared or Fisher exact test, as appropriate, to compare differences in each variable between different groups. Multivariate linear regression models were used to assess the relationship between various dependent and independent variables by adjusting for all independent variables with a *p* value < 0.1 in univariate analyses. Paired *t*-tests were used to compare variables within individuals prior to and 24 weeks after anti-HCV therapy. Levels were logarithmically transformed and used for statistical analyses, as indicated. Statistical significance was defined at the 5% level, based on two-tailed tests of the null hypothesis.

### 2.6. Informed Consent

Written informed consent was obtained from each patient. The study protocol conformed to the ethical guidelines of the 1975 Declaration of Helsinki and was approved by the hospital institutional review board.

## 3. Results

### 3.1. Baseline Characteristics

Compared with those who did not achieve SVR, lower HCV RNA level, HOMA-IR score, and G1 HCV infection and cirrhosis prevalence rates, as well as higher platelet counts and interferon-λ3 (IFNL3) CC genotype frequency were observed in SVR patients. In contrast, pre-therapy leptin, adiponectin and PAI-1 levels and L/A ratios were similar in patients with or without SVR. The pre-therapy demographics of the CHC patients are listed in Table 1.

### 3.2. Independent Pre-Therapy Factors Associated with Pre-Therapy Leptin, Adiponectin, and PAI-1 Levels in CHC Patients

**Figure 1:**
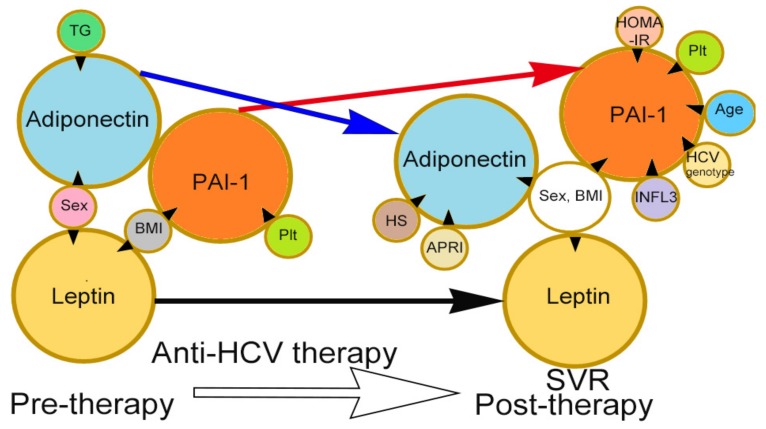
Associations between independent factors and levels of the investigated adipokines, including leptin, adiponectin, and PAI-1, at pre-therapy and 24-week post-HCV therapy stages. The tips of the black arrowheads indicate dependent factors, and the bases of the black arrowheads indicate independent factors. TGs: triglycerides; BMI: body mass index; Plt: platelet; APRI: aspartate aminotransferase to platelet ratio index; HS: hepatic steatosis; HOMA-IR: homeostasis model assessment-estimated insulin resistance; IFNL3: interferon λ3; Tx: anti-HCV therapy. Black arrow: leptin levels were unchanged after anti-HCV therapy; red arrow: PAI-1 levels were increased after anti-HCV therapy; blue arrow: adiponectin levels were decreased after anti-HCV therapy.

TC level and HOMA-IR score were independently associated with HCV RNA levels before anti-HCV therapy. Male gender was negatively associated with both leptin and adiponectin levels, and the pre-therapy BMI was positively associated with pre-therapy leptin and PAI-1 levels. Additionally, the TG level was negatively associated with adiponectin level, and the platelet count was positively associated with PAI-1 level. The PAI-1 level exhibited a borderline association with the adiponectin level (*p* = 0.05), and none of the adipokine levels were associated with the HCV RNA level (Table 2 and Figure 1).

### 3.3. Independent Post-Therapy Factors Associated with 24-Week Post-Therapy Leptin, Adiponectin and PAI-1 Levels in CHC Patients Who Achieved SVR

Gender and BMI were associated with leptin, adiponectin, and PAI-1 levels in patients who achieved SVR at 24 weeks after therapy. Additionally, APRI and steatosis were associated with the adiponectin level; age, HCV, and IFNL3 genotypes, the platelet count and the HOMA-IR score were associated with PAI-1 level (Table 3 and Figure 1).

### 3.4. Comparisons between the Pre- and Post-Therapy Levels of Each Variable in CHC Patients

**Figure 2:**
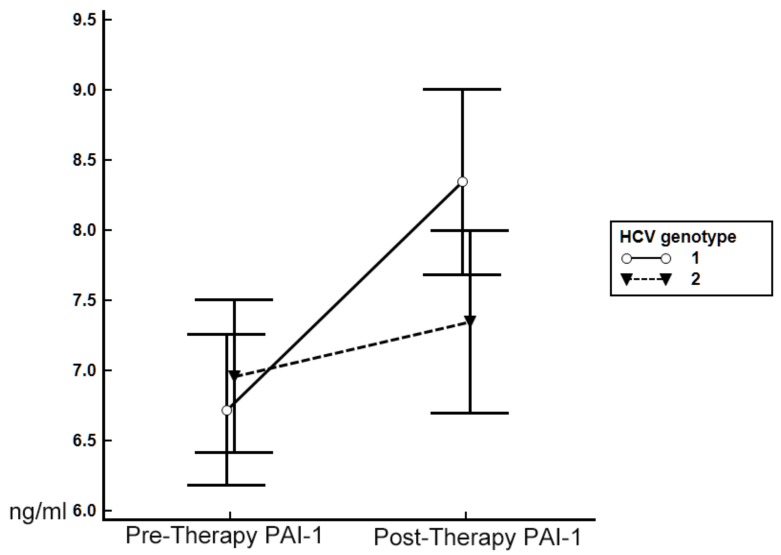
Alterations in patterns of PAI-1 levels at pre- and 24-week post-anti-HCV therapy stages in patients with SVR and stratified by HCV genotype. Pre-therapy PAI-1: pre-anti-HCV therapy levels of PAI-1 (means ± standard deviations); post-therapy PAI-1: 24-week post-anti-HCV therapy PAI-1 level. Blue line: patients with genotype 1 (G1) HCV infection; red line: patients with G2 HCV infection.

BMI decreased in CHC patients, regardless of whether they achieved SVR. However, post-therapy ALT, APRI and adiponectin levels decreased, whereas TC, TG, and PAI-1 levels increased after therapy only in patients exhibiting SVR. Regardless of the therapeutic response, leptin levels and L/A ratios remained unchanged after therapy (Table 4). Since multivariate analyses showed the HCV genotype to be associated with the post-therapy PAI-1 level in patients who achieved SVR, we stratified these patients by HCV genotype to evaluate alterations in adipokine levels. Only SVR patients with infection by G1 HCV (6.62 ± 2.91 ng/mL versus 8.43 ± 3.66 ng/mL, *p* < 0.001), but not G2 HCV (6.85 ± 3.08 ng/mL vs. 7.30 ± 3.47 ng/mL, *p* = 0.13), exhibited a significantly elevated level of PAI-1 at 24 weeks after therapy compared with the pre-therapy level (Figure 2). In contrast, the leptin level remained unchanged (G1, *p* = 0.85; G2, *p* = 0.479), and the adiponectin level decreased (G1: 9.02 ± 7.52 µg/mL versus 7.27 ± 5.40 µg/mL, *p* = 0.003; G2: 11.10 ± 7.02 µg/mL versus 8.91 ± 6.38 µg/mL, *p* < 0.001) at 24 weeks after therapy, regardless of HCV genotype.

Figure 1 summarizes the identified associations between independent factors and the investigated adipokine levels, as well as the altered levels of adipokines observed during viral clearance in CHC patients.

## 4. Discussion and Conclusions

The most compelling results of this study are as follows. (1) Prior to anti-HCV therapy, HCV genotype, TC level, and HOMA-IR score were independently associated with the HCV RNA level. Moreover, gender was associated with both leptin and adiponectin levels, and BMI was associated with leptin and PAI-1 levels. Additionally, the TG level was associated with that of adiponectin, and the platelet count was associated with the PAI-1 level. (2) Gender and BMI were associated with the levels of all investigated adipokines in patients who achieved SVR at 24 weeks after therapy. Additionally, APRI and steatosis were associated with the adiponectin level. Age, HCV, and IFNL3 genotypes, platelet count and HOMA-IR score were associated with the PAI-1 level. (3) Following anti-HCV therapy, a decrease in the adiponectin level compared to the pre-therapy level was noted in both G1 and G2 SVR patients, whereas an increased PAI-1 level was detected only in the former.

All significantly different pre-therapy variables between SVR and non-SVR patients in the current study have been reported previously [2], confirming the reliability of our data, which did not show differences in pre-therapy levels of any adipokine or the L/A ratio. Moreover, none of the pre-therapy adipokine levels were associated with the pre-therapy HCV RNA level. These results are consistent with our previous studies that focused on one adipokine, with no adjustment for other adipokines [24,25,26]. The finding of an association between the pre-therapy HCV RNA level and the TC level and HOMA-IR score confirms that HCV is a metabolic virus [2]. In general, associations of gender and BMI with the levels of investigated adipokines appear to be fundamental and more evident after viral clearance. Indeed, except for a link between adiponectin and TG level, no definite hepatic or metabolic profiles were associated with adipokine levels prior to SVR. However, after SVR, APRI (a marker of hepatic fibrosis for chronic hepatitis C [30]) and hepatic steatosis were associated with adiponectin levels, whereas the HOMA-IR score was associated with the PAI-1 level. Based on these results, adipokine levels are, to varying degrees, associated with hepatic or metabolic profiles after viral clearance, even though the effects are not obvious prior to anti-HCV therapy. Thus, HCV infection may mask or overcome interactions between metabolic/hepatic profiles and abundant adipokines.

Regardless of the adjustment for adiponectin and PAI-1 levels [25], leptin levels were consistently associated with gender and BMI and remained unchanged after viral clearance; this finding supports the crucial role of leptin in whole-body homeostasis [25]. In addition, the L/A ratio may represent a marker of adipocyte dysfunction [31] and metabolic syndrome [32] and was unchanged at 24 weeks after therapy, regardless of the therapeutic response. Conversely, although adiponectin levels were consistently associated with TG and gender before viral clearance [26], the direct association of adiponectin level with the HOMA-IR score after SVR noted in our previous study [26] disappeared after adjusting for leptin and PAI-1 levels. Prior to anti-HCV therapy, the PAI-1 level exhibited a borderline association with adiponectin levels; after SVR, the PAI-1 level was associated with the HOMA-IR score, regardless of adjustment for adiponectin and leptin levels [24]. These findings potentially indicate a higher hierarchical status of PAI-1 than adiponectin in affecting glucose metabolism once the patient is free from HCV interference. In line with the results of our previous study [24], the PAI-1 level was consistently associated with the platelet count, a finding that highlights platelets as an important source of PAI-1 [24]. Notably, the positive association between IFNL3-rs12979860 CC (a favorable SNP genotype for anti-HCV therapy [2,24,25,26,27,28,29]) and the post-therapy PAI-1 level supports the hypothesis that CHC patients who achieve SVR might have an increased risk of cardiovascular events [24]. Unexpectedly, the association of HCV genotype with PAI-1 level after SVR was evident only in the current study, which enrolled patients with both G1 and G2 HCV infections; this was not observed in our previous study, which enrolled patients with pan-genotypic HCV infections, including G1, G2, G3, G6, and mixed genotypes [24]. Based on HCV genotype-stratified analyses, increased PAI-1 after SVR was significant only in patients with G1 HCV infection, whereas altered patterns in leptin (unchanged) [25] and adiponectin (decreased) levels [26] did not change after HCV genotype stratification. The decrease in adiponectin levels observed in CHC patients after SVR was consistent with a previous case-control study showing that CHC is associated with increased serum adiponectin [33]. Our previous studies confirmed the non-hepatic origin of increases in PAI-1 observed after SVR [24] and revealed that the high PAI-1 level is linked to a poor metabolic profile [5]. Accordingly, our current finding of a G1-specific increase in PAI-1 level reiterates the observation that patients infected with G2 HCV benefit more from HCV clearance than those with G1 HCV infection due to a more favorable pattern of lipid alterations and changes in metabolic scores [27].

Since adipose tissue is the major source of adipokines [4], the main limitation of this study is the lack of pathological adipose tissue examination. Moreover, conclusions based on analyzing associated factors is an imperfect yet compromised approach to building a complete picture of adipokine-associated pathways. In addition, patatin-like phospholipase domain-containing 3 (PNPLA3) polymorphism has been shown to affect adiponectin levels in patients with non-alcoholic fatty liver disease [34,35], but not in patients with CHC [34]. PNPLA3 is not associated with the anti-HCV therapeutic response [36], and researchers have not clearly determined whether the PNPLA3 genotype is associated with altered levels of adiponectin in patients with CHC after SVR. Future studies of these adipokines in CHC patients using paired adipose tissue surveys before and at 24 weeks after therapy and an assessment of their correlations with cardiometabolic events and genetics, including the PNPLA3 genotype, as well as associated fundamental cellular or animal models may be required to elucidate the molecular basis and clinical implications of the adipokine alterations observed in patients who achieve viral clearance.

Taken together, the results show that HCV infection possibly masks the effects of abundant adipokines on hepatic or metabolic homeostasis. After viral clearance, adiponectin levels were closely associated with hepatic profiles, whereas PAI-1 was found to be in a higher hierarchical status than adiponectin with regard to affecting glucose metabolism. The genotype non-specific decrease in adiponectin levels and G1-specific increase in PAI-1 levels warrants careful follow-up of co-morbidities in patients with CHC after SVR according to viral genotype. Finally, personalized therapy and follow-up for patients with CHC may become applicable in the near future.

## Figures and Tables

**Table 1:** Baseline demographics of patients with CHC prior to anti-HCV therapy.

	Total (*n* = 450)	SVR (+) (*n* = 372)	SVR (−) (*n* = 78)	*p* values Obtained Using Student’s *t*-Test or the Chi-Squared Test
Male, *n* (%) #	256 (56.8)	212 (56.9)	44 (56.4)	0.394
Age (years)	54.01 ± 11.51	53.68 ± 11.60	55.66 ± 10.92	0.160
BMI	24.99 ± 3.73	24.84 ± 3.63	25.72 ± 4.09	0.057
HCV RNA (Log IU/mL)	5.95 ± 1.14	5.85 ± 1.18	6.45 ± 0.74	<0.001 *
HCV genotype, G1, *n* (%)	234 (52)	177 (47.5)	57 (73)	0.003 *
ALT (U/L)	94.28 ± 84.95	97.18 ± 88.37	79.80 ± 63.85	0.116
TC (mg/dL)	171.76 ± 32.22	171.53 ± 32.75	172.91 ± 29.62	0.735
TGs (mg/dL)	103.25 ± 50.15	101.21 ± 46.21	113.57 ± 66.05	0.123
Platelet count (10^3^ cells/mm)	176.89 ± 64.54	181.27 ± 57.86	156.89 ± 58.72	0.001 *
HOMA-IR	3.14 ± 4.91	2.89 ± 4.70	4.42 ± 5.73	0.043 *
Hepatic steatosis, *n* (%) ^#^	220 (48.8)	186 (50)	34 (43.5)	0.373
Liver cirrhosis, *n* (%) ^#^	117 (26)	83 (22.3)	34 (43.5)	0.001 *
APRI	1.51 ± 1.66	1.47 ± 1.63	1.74 ± 1.83	0.210
Leptin (ng/mL)	9.82 ± 10.1	9.33 ± 9.05	12.2 ± 14.1	0.267
Adiponectin (μg/mL)	9.74 ± 7.15	10.1 ± 7.48	8.04 ± 5.22	0.097
Leptin (ng/mL)/adiponectin (μg/mL) ratio	2.14 ± 3.79	2.06 ± 4.10	2.45 ± 3.00	0.603
PAI-1 (ng/mL)	6.73 ± 3.11	6.76 ± 2.98	6.56 ± 3.69	0.658
rs12979860 (CC) *n* (%) ^#^	379 (84.2)	328 (88.1)	88 (65.3)	0.013 *

SVR: sustained virological response; #: chi-squared test; * *p* < 0.05; BMI: body mass index; G: genotype; Log: logarithmic; ALT: alanine aminotransferase; TC: total cholesterol; TGs: triglycerides; HOMA-IR: homeostasis model assessment-estimated insulin resistance; APRI: aspartate aminotransferase to platelet ratio index; PAI-1: plasminogen activator inhibitor-1.

**Table 2:** Univariate and multivariate analyses of associations between pre-therapy factors and pre-therapy levels of hepatitis C virus RNA, leptin, adiponectin, and PAI-1 in all enrolled CHC patients.

	Pre-Therapy HCV RNA Level (Log IU/mL)			Pre-Therapy Leptin Level (ng/mL)			Pre-Therapy Adiponectin Level (μg/mL)			Pre-Therapy PAI-1 Level (ng/mL)		
Pre-Therapy Factors	Univariate	Multivariate		Univariate	Multivariate		Univariate	Multivariate		Univariate	Multivariate	
	*p* Values	95% CI of Estimated β (Estimated β)	*p* Values	*p* Values	95% CI of Estimated β (Estimated β)	*p* Values	*p* Values	95% CI of Estimated β (Estimated β)	*p* Values	*p* Values	95% CI of Estimated β (Estimated β)	*p* Values
Sex (Male)	0.322			<0.001 *	−12.7–−8.6 (−10.6)	<0.001 *	<0.001 *	−5.2–−1.5 (−3.4)	<0.001 *	0.094	−1.08–1.33 (0.123)	0.84
Age (years)	0.415			0.874			0.012 *	−0.03–0.13 (0.05)	0.22	<0.001 *	−0.075–0.009 (−0.033)	0.122
BMI	0.795			<0.001 *	1.09–1.70 (1.39)	<0.001 *	<0.001 *	−0.48–0.038 (−0.222)	0.094	<0.001 *	0.041–0.37 (0.205)	0.015 *
HCV RNA (Log IU/mL)	NA	NA		0.231			0.887			0.200		
HCV genotype	<0.001 *	−0.59–0.25 (−0.42)	<0.001 *	0.356			0.074	−0.1–3.5 (1.7)	0.172	0.064	−0.425–1.44 (0.509)	0.284
ALT (U/L)	0.631			0.468			0.607			0.288		
TC (mg/dL)	0.027 *	0.000–0.005 (0.003)	0.036 *	0.839			0.312			0.86		
TGs (mg/dL)	0.375			0.341			0.028 *	−0.036–−0.003 (−0.017)	0.045 *	0.095	−0.002–0.018 (0.008)	0.113
Platelet count (10^3^ cells/mm)	0.419			0.659			0.008 *	−0.027–0.005 (−0.011)	0.185	<0.001 *	0.005–0.026 (0.015)	0.005 *
HOMA-IR	0.063	0.001–0.03 (0.015)	0.04 *	0.231			0.03 *	-0.254–0.046 (−0.104)	0.171	0.909		
Hepatic steatosis (Yes)	0.837			0.001 *	−0.93–3.36 (1.22)	0.265	0.01 *	−4.01–−0.014 (−0.302)	0.052	0.009 *	−1.14–0.985 (−0.078)	0.885
Cirrhosis (Yes)	0.213			0.577			0.76			0.005 *	−1.23–1.56 (0.167)	0.813
APRI	0.496			0.489			0.213			0.012 *	−0.163–0.435 (0.136)	0.371
Leptin (ng/mL)	0.231			NA			0.313			0.015 *	−0.048–0.181 (0.017)	0.616
Adiponectin (μg/mL)	0.887			0.313			NA	NA	NA	<0.001 *	−0.142–0.007 (−0.067)	0.076
Leptin (ng/mL)	0.207			NA#			NA ^#^			<0.001 *	NA ^#^	NA ^#^
adiponectin (μg/mL) ratio												
PAI-1 (ng/mL)	0.2			0.015*	−0.19~0.45 (0.126)	0.442	<0.001 *	−0.603~0.000 (−0.302)	0.05	NA	NA	NA
rs12979860 (CC genotype)	0.246			0.636			0.743			0.236		

PAI-1: plasminogen activator inhibitor-1; Log: logarithmic; CI: confidence interval; *: *p* < 0.05; NA: not accessible; BMI: body mass index; G: genotype; ALT: alanine aminotransferase; TC: total cholesterol; TGs: triglycerides; HOMA-IR: homeostasis model assessment-estimated insulin resistance; APRI: aspartate aminotransferase to platelet ratio index; # these values are not listed as independent factors in univariate or multivariate analyses because they are highly dependent on leptin and adiponectin levels, which are independent factors.

**Table 3:** Univariate and multivariate analyses of associations between post-therapy factors and post-therapy levels of leptin, adiponectin, and PAI-1 in CHC patients after SVR.

	Post-therapy Leptin Level (ng/mL)			Post-therapy Adiponectin Level (μg/mL)			Post-therapy PAI-1 Level (ng/mL)		
	Univariate	Multivariate		Univariate	Multivariate		Univariate	Multivariate	
Post-Therapy Factors	*p* Values	95% CI of Estimated β (Estimated β)	*p* Values	*p* Values	95% CI of Estimated β (Estimated β)	*p* Values	*p* Values	95% CI of Estimated β (Estimated β)	*p* Values
Sex (Male)	<0.001 *	−18.9–−10.3 (−15.2)	<0.001 *	<0.001 *	−5.7–−2.6 (−4.2)	0.001 *	0.008 *	0.8–1.86 (0.97)	0.033 *
Age	0.317			0.223			<0.001 *	−0.12–−0.04 (−0.08)	<0.001 *
BMI	<0.001 *	1.31–2.52 (1.88)	<0.001 *	<0.001 *	−0.53–−0.05 (−0.29)	0.017 *	<0.001 *	0.06–0.308 (0.186)	0.003 *
HCV genotype	0.193			0.545			0.034 *	−1.6–−0.005 (−0.81)	0.049 *
ALT (U/L)	0.958			0.07	−0.08–0.04 (−0.02)	0.515	0.471		
TC (mg/dL)	0.079	−0.003–0.09 (0.05)	0.066	0.201			0.246		
TGs (mg/dL)	0.549			<0.001 *	−0.02–0.008 (−0.006)	0.382	<0.001 *	−0.005–0.001 (0.002)	0.551
Platelet counts (10^3^ cells/mm)	0.523			0.448			<0.001 *	0.014–0.035 (0.025)	<0.001 *
HOMA-IR	0.089	−0.32–0.69 (0.184)	0.473	0.006 *	−0.46–−0.003 (−0.22)	0.053	0.039 *	0.05–0.30 (0.182)	0.004 *
APRI	0.564			0.015 *	0.11–5.59 (2.85)	0.042 *	<0.001 *	−0.77–2.4 (0.814)	0.313
Hepatic steatosis (Yes)	0.069	−2.9–4.6 (0.884)	0.656	<0.001 *	−3.8–−0.56 (−2.2)	0.008 *	0.008 *	−0.29–1.41 (0.56)	0.794
Cirrhosis (Yes)	0.315			0.419			<0.001 *	−2.2–0.19 (−0.98)	0.100
Leptin (ng/mL)	NA	NA	NA	0.29			0.781		
Adiponectin(μg/mL)	0.29			NA	NA	NA	0.008 *	−0.05–0.09 (0.02)	0.592
Leptin (ng/mL)/adiponectin (μg/mL) ratio	NA ^#^			NA ^#^			0.044 *	NA ^#^	NA ^#^
PAI-1 (ng/mL)	0.781			0.008 *	−0.18–0.28 (0.053)	0.65	NA	NA	NA
rs12979860 (CC)	0.853			0.915			0.076	0.46–2.4 (1.49)	0.004 *

PAI-1: plasminogen activator inhibitor-1; SVR: sustained virological response; CI: confidence interval; *: *p* < 0.05, NA: not accessible; BMI: body mass index; ALT: alanine aminotransferase; TC: total cholesterol; TGs: triglycerides; HOMA-IR: homeostasis model assessment-estimated insulin resistance; APRI: aspartate aminotransferase to platelet ratio index; # these values are not listed as independent factors in univariate or multivariate analyses because they are highly dependent on leptin and adiponectin levels, which are independent factors.

**Table 4:** Comparison of the pre- and 24-week post-therapy variables in CHC patients stratified by therapeutic response.

	SVR (+) (*n* = 372)		Paired *t*-Test *p* Values	SVR (−) (*n* = 78)		Paired *t*-Test *p* Values
Factors	Pre-TherapyValue	Post-TherapyValue		Pre-TherapyValue	Post-TherapyValue	
BMI	24.84 ± 3.63	24.35 ± 3.51	<0.001 *	25.72 ± 4.09	24.87 ± 3.62	<0.001 *
ALT (U/L)	97.18 ± 88.37	20.0 ± 10.5	<0.001 *	79.80 ± 63.85	63.7 ± 43.3	0.151
TC (mg/dL)	171.53 ± 32.75	184.28 ± 37.39	<0.001 *	172.91 ± 29.62	174.29 ± 36.12	0.7021
TGs (mg/dL)	101.21 ± 46.21	120.54 ± 74.75	<0.001 *	113.57 ± 66.05	102.77 ± 42.88	0.059
Platelet count (10^3^ cells/mm)	181.27 ± 57.86	184.10 ± 55.68	0.243	156.89 ± 58.72	149.40 ± 54.55	0.179
HOMA-IR	2.89 ± 4.70	2.83 ± 3.96	0.5493	4.42 ± 5.73	5.40 ± 11.55	0.7332
APRI	1.47 ± 1.63	0.418 ± 0.297	<0.001 *	1.74 ± 1.83	1.28 ± 0.929	0.162
Hepatic steatosis (Yes), *n* (%)	186 (50)	193 (51.8)	0.499	34 (43.5)	37 (47.4)	0.443
Cirrhosis (Yes), *n* (%)	83 (22.3)	86 (23)	0.504	34 (43.5)	39 (50)	0.058
Leptin (ng/mL)	9.33 ± 9.05	9.61 ± 8.77	0.15	12.2 ± 14.1	10.1 ± 12.5	0.187
Adiponectin (μg/mL)	10.1 ± 7.48	8.14 ± 5.09	0.003*	8.04 ± 5.16	8.77 ± 6.32	0.473
Leptin (ng/mL)/adiponectin (μg/mL) ratio	1.96 ± 4.07	2.26 ± 3.57	0.188	2.44 ± 2.96	3.02 ± 6.43	0.506
PAI-1 (ng/mL)	6.76 ± 2.98	9.08 ± 4.43	0.003*	6.56 ± 3.69	6.45 ± 4.42	0.9355

SVR: sustained virological response; *: *p* < 0.05; BMI: body mass index; ALT: alanine aminotransferase; TC: total cholesterol; TGs: triglycerides; HOMA-IR: homeostasis model assessment-estimated insulin resistance; APRI: aspartate aminotransferase to platelet ratio index; PAI-1: plasminogen activator inhibitor-1.
